# Urine Metabolites and Antioxidant Effect after Oral Intake of Date (*Phoenix dactylifera* L.) Seeds-Based Products (Powder, Bread and Extract) by Human

**DOI:** 10.3390/nu11102489

**Published:** 2019-10-16

**Authors:** Carine Platat, Serene Hillary, Francisco A. Tomas-Barberan, J. Alberto Martinez-Blazquez, Fatima Al-Meqbali, Usama Souka, Suleiman Al-Hammadi, Wissam Ibrahim

**Affiliations:** 1 Department of Food, Nutrition and Health, College of Food and Agriculture, United Arab Emirates University, Al Ain PO Box 15551, UAE; 201590078@uaeu.ac.ae (S.H.); fatmadiab@uaeu.ac.ae (F.A.-M.); usamasouka@uaeu.ac.ae (U.S.); wibrahim@uaeu.ac.ae (W.I.); 2 Quality, Safety and Bioactivity of Plant Foods, CEBAS-CSIC, PO Box 164, Espinardo, 30100 Murcia, Spain; fatomas@cebas.csic.es (F.A.T.-B.); albertomartinezblazquez@gmail.com (J.A.M.-B.); 3 Department of Pediatrics, College of Medicine and Health Sciences, United Arab Emirates University, Al Ain P.O. Box 15551, UAE; suleiman.alhammadi@uaeu.ac.ae

**Keywords:** date seeds, oxidative damages, antioxidant, human, urine metabolites, polyphenols, proanthocyanidins, UPLC-ESI-QTOF(MS/MS)

## Abstract

A cross-over study was conducted in 16 healthy adult volunteers to describe the urinary excretion of polyphenols from date seeds and investigate the antioxidant effect after consumption of different doses of date seeds powder (DSP), bread (DSB) and extract (DSE). After 12 h of fasting, one of the six treatments (0.25 g and 0.5 g/kg bodyweight DSP, 360 g of 10% and 15% DSB, 30 mg and 60 mg/kg bodyweight DSE) was provided along with breakfast, with a two weeks wash-out period between 2 consecutive treatments. Blood was drawn at baseline, 1, 2, 8 and 24 h post intake. Urine was collected at baseline, 3, 8, and 24 h post intake. An abundant release of polyphenols was detected in urine within the 0–3 h post intake, reached a peak at 8 h, then decreased with polyphenols still being detected up to 24 h post intake. The antioxidant defence system, as measured by reduced glutathione (GSH), was strengthened as soon as 1 h and up to 8 h post intake. Markers of protein and lipid oxidative damages were reduced from 1 h and up to 8 and 24 h post intake, respectively. This supports an antioxidant effect of date seeds products in humans, most probably due to their polyphenols.

## 1. Introduction

Due to a continuous and worrying increase, chronic diseases including cardiovascular diseases, diabetes and cancer constitute a substantial public health concern worldwide. Oxidative stress and free radicals are playing a vital role in the development of these diseases by inducing damages on cells and macromolecules. The human body has integrated defence systems, including glutathione. They are usually effective in protecting against oxidative damages. However, overwhelming them with an excess of free radicals can cause diseases. Due to their antioxidant properties, some natural compounds can provide a second protective barrier [1]. Among them, polyphenols are some of the most potent ones. They are found in enormous amounts in fruits and vegetables. They demonstrate many protective effects against cancer, inflammation and ageing [2], explaining why the World Health Organisation (WHO) is recommending a daily consumption of a variety of fruits and vegetables [3]. In the past years, the by-products of the manufacturing of fruits and vegetables, and primarily the seeds, drew the attention due to their high content in polyphenols. Indeed, seeds from fruits like grape, pomegranate, pumpkin, cherry, mango, currant or kiwi are listed among the highest sources of polyphenols [4,5,6,7]. Noticeably, the multiple biological properties which were associated with them were attributed mainly to their polyphenolic content [7,8,9,10,11].

Date (*Phoenix dactylifera* L.) is a fruit which is primarily produced in the Middle East region. It is not only consumed locally but in other parts of the world as well. Date seeds constitute 6%–15% of the total weight of the ripe date depending on variety and grade quality. They are being wasted in large quantities or used mainly for animal (camels, cattle, sheep, and poultry) feed. Interestingly, date seeds possess excellent nutritional quality. The determination of the macro- and micro-nutrient profiles of various varieties of date seeds revealed a high amount of fibre (67.6–74.2 g/100 g) and considerable amounts of some minerals, vitamins, lipids, and protein [12,13]. Additionally, date seeds were shown to be particularly rich in polyphenols [12,14,15]. Depending on the variety, a concentration in polyphenols between 1864.82 and 4768.87 mg gallic acid equivalents/100 g was reported [12]. A detailed exploration of the polyphenolic compounds identified flavan-3-ols, especially catechins and epicatechins, as the most abundant ones in date seeds with up to 50.18 g/kg flavan-3-ols in the *Khalas* variety [15]. Besides, two pre-clinical studies indicated that date seeds extract was able to reduce the oxidation of macromolecules in rats’ serum and organs and strengthen the antioxidant defence system of the animals, with doses of 240 mg and 480 mg/kg diet [16,17]. With such results, date seeds, among the large variety of fruit seeds, represent a right candidate for the use as a functional ingredient.

However, the level of availability of the polyphenols in the human body, also called bioavailability, is conditioning the expression of their antioxidant effects. Usually, native compounds found in plants undergo conversions in the digestive tract and can be extensively metabolized, leading to the release of completely different bioactive polyphenols. Therefore, to confirm the in vivo antioxidant potential of any natural compound, it is necessary to determine how all these transformations affect them and their bioavailability. Besides, in the ultimate purpose of functional food development, it is also essential to take into account the possible interactions of polyphenols with the food matrix that may alter polyphenols’ health effects [18]. Very few attempted functional food products have been developed and tested so far in clinical studies. Bread represents a matrix of choice for easy incorporation of seeds powder. Besides, it is an important food component of the diet in the Middle East. Results on a grape seeds bread and on a date seeds pita bread have already been reported [19,20]. Interestingly, the 10% date seeds pita bread and the 5% grape seeds bread were presenting similar content in catechins and both significantly improved the antioxidant capacity of the bread. So far, experimentation in healthy humans is still missing.

The goals of this present work are to study the excretion of metabolites of polyphenols in urine, after consumption of one dose of date seeds powder, date seeds extract or date seeds bread by healthy human volunteers and to identify related health effects, especially an antioxidant activity. This will provide an insight into the real bioavailability of date seeds polyphenols and demonstrate the health benefit of consuming date seeds products.

## 2. Materials and Methods

### 2.1. Study Design and Participants

A cross-over design, with six arms, was conducted in 16 healthy adults, including eight women and eight men. They were included after an initial screening visit during which exclusion and inclusion criteria were checked. Inclusion criteria for the study were: age between 18 and 55 years, normal weight status (body mass index between 18.5 and 24.9 kg/m^2^), no metabolic or intestinal disorder, no history of gastrointestinal surgery (except appendectomy), under no prescription medication, no alcohol consumption, non-smoking, not taken antibiotics during the 6 months preceding inclusion in the study, and no blood donation during the month preceding inclusion in the study. The whole study was conducted in the Ain Al Khaleej Hospital, Al Ain, United Arab Emirates.

In that study, three date seed products, date seed powder (DSP), date seed bread (DSB) and date seed extract (DSE), were considered. Two different doses of each product were tested, either a total of six treatments, with each subject receiving a single dose of each treatment (Table 1). Each subject completed six visits (one visit per treatment) of 1 day at the hospital. At each visit, subjects were asked to come to the hospital at 7 am in a fasting state to receive the treatment along with their breakfast. Two consecutive visits were separated by a two weeks wash-out period. The order for the treatments was the same for all the subjects. At each visit, subjects stayed at the hospital during the 8 h following the ingestion of the treatment, then left for the night and came back at 7 am the day after. Two days before each visit, subjects were asked to not perform any moderate or vigorous physical activity and to refrain from eating any polyphenols-rich products, including dates. A detailed list of food products to avoid was provided to the subjects. In addition to a written information sheet, oral explanations were provided to each subject about the aim and procedures of the trial. Subjects signed a written informed consent form before any data collection. The protocol was approved by the Al Ain Medical District Human Research Ethics Committee, on 16 December 2014 (protocol number 14/70).

### 2.2. Date Seed Products

Date palm (*Phoenix dactylifera* L.) seeds of the Khalas variety were used in this trial. They were provided by the Al Foah Company-Emirates Dates factory, Al-Ain, United Arab Emirates. Dates were collected randomly from tamr (fully ripe dates) batches at the end of the season with no preference to size, colour, appearance, or firmness. Date seed powder (DSP) was prepared by milling cleaned and dried date seeds. The powder was sieved by a BZS 200 sieve machine and particles of less than 300 microns were used in the study as treatments: DSP1 and DSP2 (Table 1). Date seed bread (DSB), 10% and 15%, was baked in-house using the procedure described by Platat et al. [20]. Date seed extract (DSE) was prepared by extracting the DSP with an ethanol:water (1:1) solution. The extract was filtered using Whatman filter paper, was reduced under nitrogen, and vacuum-dried to yield the extract. The polyphenolic profile of DSP, DSB, and DSE have already been determined earlier [21]. Based on this data, the total polyphenols content of the two doses of powder, bread, and extract were 0.74 g, 1.48 g, 1.79 g, 2.69 g, 0.075 g, and 0.15 g, respectively (Table 1).

### 2.3. Treatments

A total of 6 date seed products-based treatments were tested with each participant receiving a single dose of each treatment (Table 1). DSP, 0.25 g and 0.5 g/kg Body Weight (BW) (DSP1 and DSP2), was provided as a beverage after solubilization into water. A non-nutritious mango flavouring agent was added for the palatability of the beverage. The final volume of beverage to be ingested by the subjects was 330 mL. For each DSB, 10% and 15% DSP (DSB1 and DSB2), six loaves, each around 60 g, were consumed. DSE is like a paste that was provided at the concentrations of 30 and 60 mg/kg BW (DSE1 and DSE2). Treatments were provided along with a breakfast, which was covering 50% of the individual daily energy needs as calculated by the Harris–Benedict equation and was containing 10%–15% of fat and 15%–20% protein. DSP1, DSP2, DSE1, and DSE2 were offered with regular Arabic bread and butter. DSB1 and DSB2 were consumed with butter and water. Subjects were requested to finish the entire breakfast and for the next 8 h, only water was allowed. If a subject reported any food consumption within the past 10 h preceding their arrival at the hospital, the experiment was cancelled and rescheduled as soon as possible.

### 2.4. Blood and Urine Collection

Blood was collected at different time points on serum separating tubes and ethylenediamine tetraacetic acid (EDTA) tubes by phlebotomist at the hospital, then promptly centrifuged. Plasma and serum were stored at −80 °C. The points which are of interest for this study are baseline (fasting status, before date seeds-based products ingestion), 1, 2, 8 and 24 h after ingestion. Urine was collected at baseline and 24 h. In-between, cumulative urine was kept in a hermetic and labelled container in the fridge, over the post-ingestion periods 0–3 h, 3–8 h and 8–24 h. All samples were stored at −80 °C before analysis.

### 2.5. Biochemical Parameters

Glucose, total cholesterol, Low Density Lipoprotein (LDL)-cholesterol, High Density Lipoprotein (HDL)-cholesterol, triglycerides, creatinine, albumin, urea, creatine kinase, lactate dehydrogenase, gamma-glutamyltransferase (GGT), aspartate transaminase (AST), alanine transaminase (ALT) and alkaline phosphatase (ALP) were measured in serum using enzymatic colorimetric methods on Roche/Hitachi Cobas C systems (Integra 400 Plus, Mannheim, Germany). NCEP guidelines were used as a reference for the lipid profile (National Cholesterol Education Program, 2002). For the other biochemical parameters, normal ranges as provided by the manufacturer were considered. Total protein content was measured following the method of Lowry et al. [22].

### 2.6. Urine Analysis of Polyphenols Metabolites

An ultra-performance liquid chromatography-electrospray ionization/quadrupole-time-of-flight (UPLC-ESI-QTOF) mass spectrometry (MS) was employed to analyse the polyphenolic metabolites in urine using a method described earlier [23]. Briefly, 1 mL of lyophilised urine sample was reconstituted with QTOF grade water containing 1 ppm rutin as internal standard and filtered using 0.22 µm syringe filter, and injected in Agilent 1290 Infinity UPLC system coupled to a 6550 Accurate-Mass QTOF MS system (Agilent Technologies, Waldbronn, Germany). The mobile phases were water with 0.1% formic acid and acetonitrile with 0.1% formic acid. The injection volume was 3 µL. Poroshell 120 EC-C18 column (3 × 100 mm, 2.7 um) (Agilent Technologies) was used for the experiment and the column was maintained at 30 °C. The flow rate was 0.4 mL/min and the following gradient was set up for the analysis. The linear gradient started with 5% of solvent B in A, reaching 18% solvent B at 7 min, 28% at 17 min, 50% at 22 min, and 90% at 27 min, which was maintained up to 28 min. Initial conditions were re-established after 29 min. The following conditions were maintained for ESI: flow rate 0.4 mL/min, gas temperature 300 °C, drying gas 11 L/min, nebuliser pressure 45 psi, sheath gas temperature 400 °C, and sheath gas flow 12 L/min. The spectra were acquired in scan MS mode with m/z range of 100–1100 in negative polarity mode. The acquisition rate was 1.5 spectra/s. The MS/MS parameters were set as m/z range of 50–800 with a retention time window of 1 min and collision energy from 20 to 40 V and an acquisition rate of 4 spectra/s. The data processing was carried out with MassHunter Qualitative Analysis software (version B.06.00, Agilent Technologies, Waldbronn, Germany).

### 2.7. Biomarkers of the Oxidative Status

Measurements were done at baseline, 1, 2, 8 and 24 h after ingestion. Glutathione reduced form (GSH) was measured spectrophotometrically by using the method described earlier [24], which measures the non-protein sulfhydryl groups (NP-SH) by spectroscopy using 5,5′-dithiobis-(2-nitrobenzoic acid) (DTNB) reagent. Briefly, 100 µL of plasma was precipitated with 20% trichloroacetic acid (TCA) (vol/vol) and 100 µL supernatant was retrieved after centrifuging the samples at 10,000× *g* for 10 min. The 100 µL of 0.2 M tris buffer (pH 8.9, substituted with 0.2 M ethylenediamine tetraacetic acid (EDTA) solution in the ratio of 1 EDTA:10 Tris) was added to the supernatant followed by 5µL of 0.01 M DTNB reagent (wt/vol in methanol). The absorbance of the samples was read against blank at 412 nm and the results were calculated using the formula below:

$$GSH\;in\;nmol/ml=\left(\left(\frac{\;Absorbance}{13.6\;\times 0.96945}\right)\times 305\right)\times 200$$

Malondialdehyde (MDA), which is a lipid peroxidation product, was measured using the modified thiobarbituric acid reactive substances (TBARs) procedure [25]. Briefly, plasma samples were precipitated using 20% TCA and centrifuged at a high speed to retrieve the supernatant. The supernatant was incubated with 0.4% TBA (Thiobarbituric acid, w/v in 0.2 N HCl) at 60 °C for one hour for the formation of TBA-MDA adducts. The adducts formed in the sample was measured by the Breeze HPLC System (Waters, USA). The mobile phase was methanol:0.05 M KH_2_ PO_4_ buffer, pH 6.8 (40:60, v/v) containing 0.2% (v/v) triethanolamine. Xterra MS C18 reverse phase column of 5 μm pore size was used for the analysis. The column temperature was 35 °C and the flow rate was 1 mL/min with an injection volume of 20 μL. The fluorescence detection wavelength was set at 532 nm (excitation) and 553 nm (emission). All samples were analysed in duplicate. Protein-bound carbonyls (PC) was used to assess the extent of protein oxidation. It was determined spectrophotometrically at 370 nm by the 2,4-dinitrophenylhydrazine method described by Castegna et al. [26].

### 2.8. Dietary Assessment

A food frequency questionnaire was administered at screening, mainly to estimate the polyphenol intake of each subject. It was adapted from the questionnaire developed for Iranian adults by Mirmiran et al. [27]. The average daily consumption of polyphenols was estimated by using the polyphenols content of the food products, as indicated in the Phenol-Explorer database. Database URL: http://www.phenol-explorer.eu.

### 2.9. Statistical Analysis

SPSS (v24) was used to perform statistical analysis. Means ± standard deviation (SD) were calculated. ANOVA was used for gender comparisons. When no gender difference was detected, results for the whole sample will be presented. Results for biochemical parameters at baseline and 24 h were compared by using paired t-tests. An ANOVA model with repeated measures was performed to compare the values of GSH, MDA and PC at baseline, 1, 2, 8 and 24 h. When the assumption of sphericity was violated, the greenhouse-Geisser correction was considered. Post hoc Bonferroni tests were used to determine which specific means differed. Gender and average daily polyphenols consumption in tertiles were introduced as between-subject factors. Data from urinary excretion of metabolites of date seeds were analysed using the same parameters. The post hoc Bonferroni test was used to compare data at baseline (0 h), 0–3 h, 3–8 h and 8–24 h. Comparisons were also made between the two doses of DSP, DSB and DSE. GraphPad Prism version 8.1.2 was used to build figures for the manuscript.

## 3. Results

### 3.1. Participants and Polyphenols Consumption

Sixteen subjects, eight women and eight men were initially included. One subject decided to drop out after the first two experiments and was immediately replaced by a subject from the same gender. After blood samples analysis, abnormally high levels of fasting glucose and dyslipidemia were detected for two subjects. Even though fasting status was orally checked by the research team and the nurse before baseline blood collection, it cannot be excluded that subjects were not fasting. Consequently, they were excluded from the analysis. Also, they were advised to visit their regular physician to follow-up with their glucose level and lipid profile. Finally, 14 subjects, eight women and six men, were included in the statistical analysis. They were between the ages of 25.43 ± 5.11 years on average and all had a healthy weight status. Their average consumption of polyphenols was 3.02 ± 1.93 g/d (Table 2).

### 3.2. Biochemical Assessment

Before consumption of any date seeds products at DSP1 baseline, all subjects had a normal fasting glucose and lipid profile. Only the HDL-Cholesterol level was below the NCEP recommended level for both genders (Table 3). Twenty-four hours after ingestion of date seeds powder and date seeds bread, these parameters did not significantly change. With DSE1, an increase of glucose, total cholesterol, and LDL-cholesterol levels were observed. However, the values at 24 h remained within the normal range and no similar change was detected with a higher dose of the date seeds extract (DSE2). Globally, biomarkers of tissue injury and inflammation were kept within normal ranges in all of the treatments (Table 4). Constant changes were detected, but parameters never reached abnormal values.

### 3.3. Urinary Metabolites

Twenty predominant metabolites were detected in the urine samples following ingestion of all date seed treatments (Table 5). Extracted Ion Chromatogram was retrieved for over 53 known metabolites of flavan-3-ols and 20 were identified by a mean score of 90 or more. The compounds were confirmed by their formula, (M-H)- and MS-spectrum. The predominant metabolites include two isomers of hydroxybenzoic acid, two isomers of protocatechuic acid, two isomers of vanillic acid, vanillic acid sulphate, ferulic acid sulphate, and methyl epicatechin sulphate. We also detected the prevalent metabolites of proanthocyanidins, such as 3,4-dihydroxyphenyl valerolactone and its glucuronide and sulphate. Significant level aromatic acids such as 3-hydroxyphenyl acetic acid, 3-hydroxyphenyl propanoic acid, 3-hydroxyphenyl valeric acid, 3,4-hydroxyphenyl valeric acid and its sulphate were also detected in urine. Other metabolites include hippuric acid and two isomers of hydroxy-hippuric acid.

**Figure 1:**
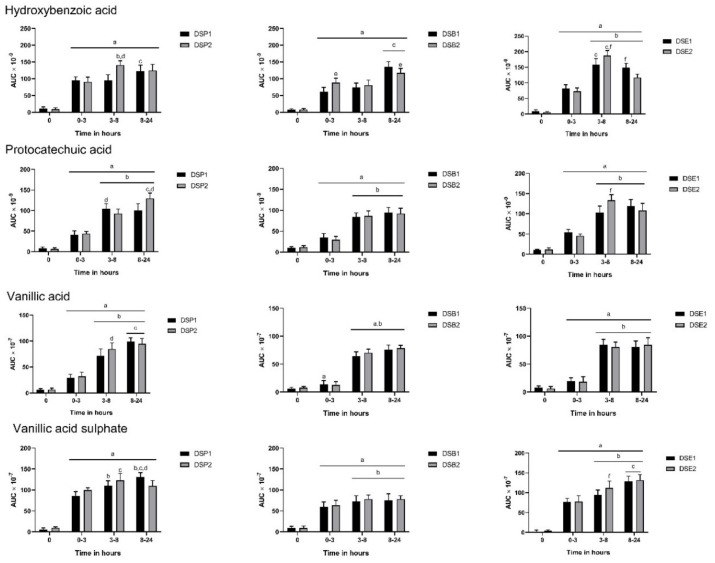
Polyphenol metabolites in urine after consumption of date seed treatments. Data were calculated as the area under curve (AUC) adjusted to total urine output at the respective time interval. Mean AUC ± SD are presented. ANOVA with multiple comparisons was performed. Statistical significance was set at *p* ≤ 0.05. ^a^ Statistically significant difference in comparison to 0 h. ^b^ Statistically significant difference in comparison to 0–3 h. ^c^ Statistically significant difference between 3–8 h and 8–24 h. ^d^ Statistically significant difference between DSP1 and DSP2. ^e^ Statistically significant difference between DSB1 and DSB2. ^f^ Statistically significant difference between DSE1 and DSE2.

A significant increase in the level of aromatic acids such as hydroxybenzoic acid, protocatechuic acid, vanillic acid and vanillic acid sulphate was observed in three hours in urine (Figure 1). Hydroxybenzoic acid was detected in urine after consumption of all six date seed treatments. We observed a significant increase in hydroxybenzoic acid levels after three hours of ingestion. The release of this metabolite was sustained up to 24 h in all subjects. Interestingly, with ingestion of the DSP2 and DSE2, the level of hydroxybenzoic acid peaked between 3–8 h. Predictably, with the higher dose of powder and extract (DSP2 and DSE2), a significant increase in hydroxybenzoic acid levels were observed at 3–8 h. We observed the similar difference in bread with supplementation of DSP2 giving higher levels of hydroxybenzoic acid at 0–3 h. Consumption of date seed products also resulted in the release of protocatechuic acid in the urine (Figure 1). Similar to hydroxybenzoic acid, we observed increased levels of protocatechuic acid up to 8 h after ingestion of the treatments, which was sustained up to 24 h. Interestingly, more protocatechuic acid was observed with DSP1 at 3–8 h than 8–24 h, but it was sustained since at 24 h, the level of protocatechuic acid was increased with DSP2. A significantly higher level of protocatechuic acid was observed with DSE2 when it peaked between 3–8 h. Urinary excretion of vanillic acid also observed constant changes (Figure 1). Vanillic acid levels peaked between 8–24 h with date seed powder treatments, however, no change between 3–8 h and 8–24 h was observed with extract and powder. The only significant difference between doses was observed with powder ingestion at 3–8 h, where DSP2 recorded higher levels of protocatechuic acid. Vanillic acid sulphate levels increased similarly to vanillic acid (Figure 1).

**Figure 2:**
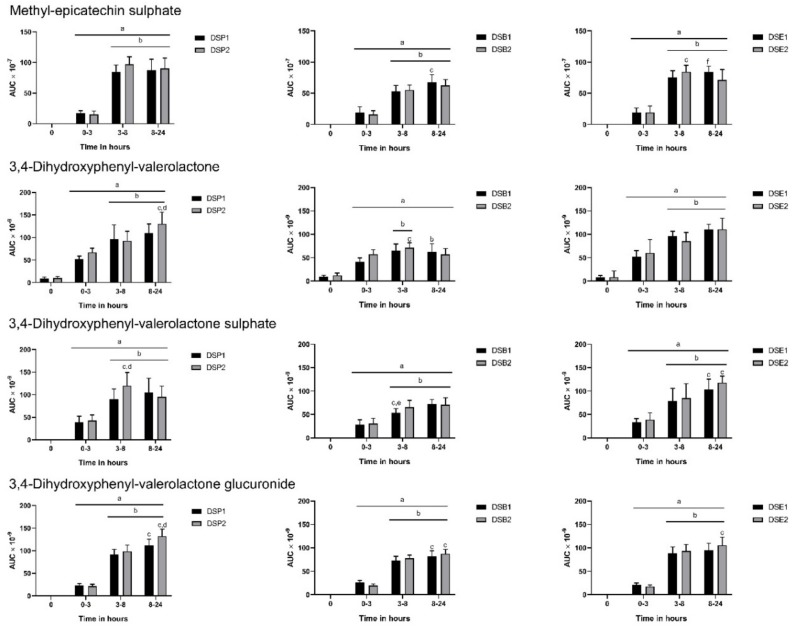
Polyphenol metabolites in urine following consumption of date seed polyphenols. Data were calculated as area under curve (AUC) adjusted to total urine output at the respective time interval. Mean AUC ± SD are presented. ANOVA with multiple comparisons was performed. Statistical significance was set at *p* ≤ 0.05. ^a^ Statistically significant difference in comparison to 0 h. ^b^ Statistically significant difference in comparison to 0–3 h. ^c^ Statistically significant difference between 3–8 h and 8–24 h. ^d^ Statistically significant difference between DSP1 and DSP2. ^e^ Statistically significant difference between DSB1 and DSB2. ^f^ Statistically significant difference between DSE1 and DSE2.

The predominant metabolites of proanthocyanidins, such as methyl-epicatechin sulphate and valerolactone compounds, were found the urine samples from all subjects (Figure 2). There was the sustained release of methyl-epicatechin sulphate by the subjects following ingestion of date seed treatments between 3–24 h. There was no difference in the doses in bread and powder, but DSE1 gave higher levels of the metabolite between 8–24 h. A similar trend in urinary excretion was maintained for all valerolactone compounds which includes 3,4-dihydroxyphenyl valerolactone, 3,4-dihydroxyphenyl valerolactone glucuronide, and 3,4-dihydroxyphenyl valerolactone sulphate. 3,4-dihydroxyphenyl-valerolactone and its glucuronide was significantly increased between 8–24 h with consumption of DSP2. No such differences were observed with the bread and powder treatments for this metabolite. In the case of 3,4-dihydroxyphenyl-valerolactone sulphate, between 3–8 h, we observed higher levels with DSP2 and DSB1.

**Figure 3:**
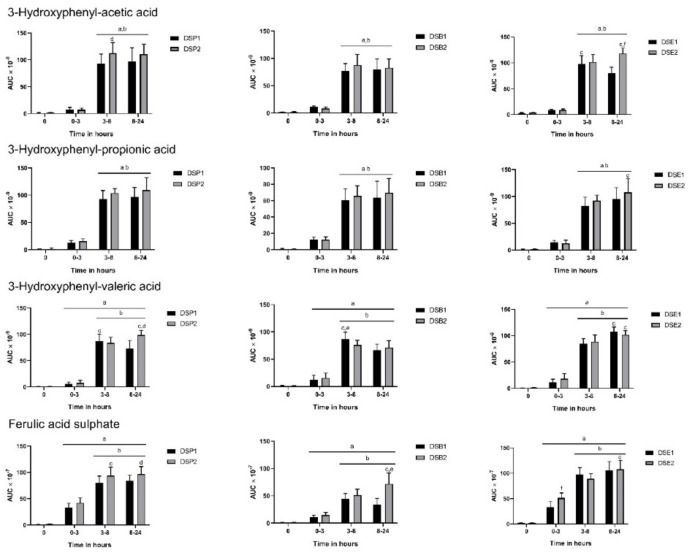
Polyphenol metabolites in urine following consumption of date seed treatments. Data were calculated as area under curve (AUC) adjusted to total urine output at respective time interval. Mean AUC ± SD are presented. ANOVA with multiple comparisons was performed. Statistical significance was set at *p* ≤ 0.05. ^a^ Statistically significant difference in comparison to 0 h. ^b^ Statistically significant difference in comparison to 0–3 h. ^c^ Statistically significant difference between 3–8 h and 8–24 h. ^d^ Statistically significant difference between DSP1 and DSP2. ^e^ Statistically significant difference between DSB1 and DSB2. ^f^ Statistically significant difference between DSE1 and DSE2.

**Figure 4:**
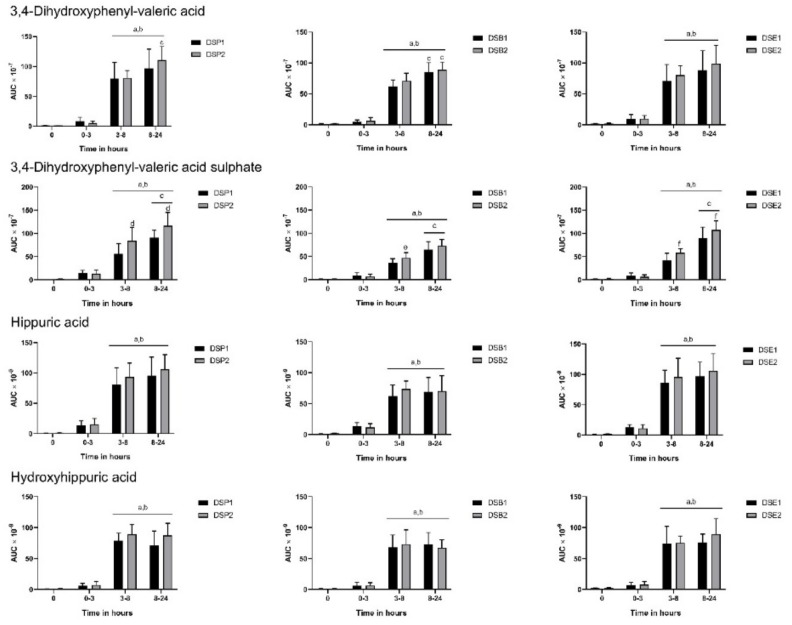
Polyphenol metabolites in urine following consumption of date seed treatments. Data were calculated as area under curve (AUC) adjusted to total urine output at the respective time interval. Mean AUC ± SD are presented. ANOVA with multiple comparisons was performed. Statistical significance was set at *p* ≤ 0.05. ^a^ Statistically significant difference in comparison to 0 h. ^b^ Statistically significant difference in comparison to 0–3 h. ^c^ Statistically significant difference between 3–8 h and 8–24 h. ^d^ Statistically significant difference between DSP1 and DSP2. ^e^ Statistically significant difference between DSB1 and DSB2. ^f^ Statistically significant difference between DSE1 and DSE2.

All three hydroxyphenyl acids, 3-hydroxyphenyl acetic acid, 3-hydroxyphenyl propionic acid, and 3-hydroxyphenyl valeric acid, released in the gut were predictably increased from 3 h to 24 h in urine following consumption of date seed treatments (Figure 3). Dose differences were observed with DSP2 in urinary excretion of 3-hydroxyphenyl-acetic acid and valeric acid. We observed a similar difference in dose with extract in 3-hydroxyphenyl-acetic acid alone, with DSE2 giving higher levels of the metabolite. Similarly, ferulic acid sulphate was also increasing with time (Figure 3). Ferulic acid sulphate levels were increased with higher doses of extract and bread in our study. Other aromatic acids, such as 3,4-dihydroxyphenyl-valeric acid, 3,4-dihydroxyphenyl valeric acid sulphate, hippuric acid, and hydroxyhippuric acid also observed constant changes with time (Figure 4). Most of these compounds originating in the colon through the action of gut microbes increased in levels predictably between 3–8 h and were sustained up to 24 h. No significant difference was observed between 3–8 h and 8–24 h or between doses at these time intervals in the excretion of hippuric acid and hydroxyhippuric acid (Figure 4). However, in the case of 3,4-dihydroxyphenyl-valerolactone sulphate, dose level differences could be observed, with ingestion of DSP2, DSB2, and DSE2 giving higher levels of the metabolite.

### 3.4. Biomarkers of Oxidative Stress

**Figure 5:**
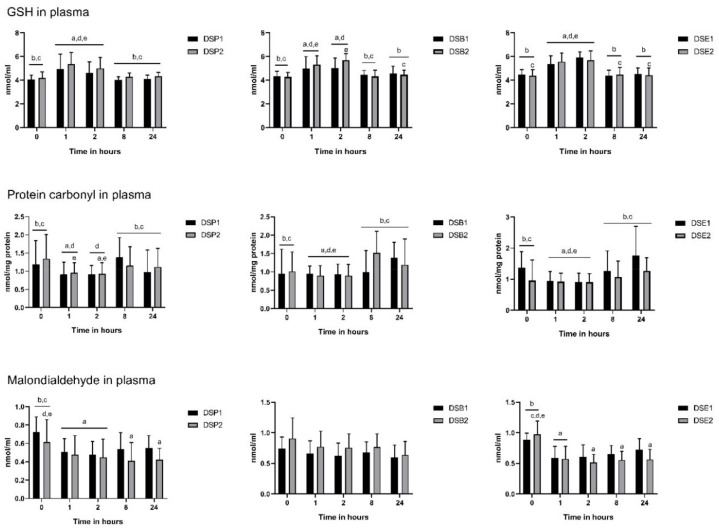
Oxidative stress biomarker levels at baseline (0 h), 1, 2, 8 and 24 h after date seed treatments ingestion. Means ± SD are presented. ANOVA with repeated measures was performed. Gender and polyphenols consumption tertiles were added into the model as a between-subjects factor. Statistical significance was set at *p* ≤ 0.05. ^a^ Statistically significant difference with baseline; ^b^ Statistically significant difference with time point 1 h; ^c^ Statistically significant difference with time point 2 h; ^d^ Statistically significant difference with time point 8 h; ^e^ Statistically significant difference with time point 24 h.

**Figure 6:**
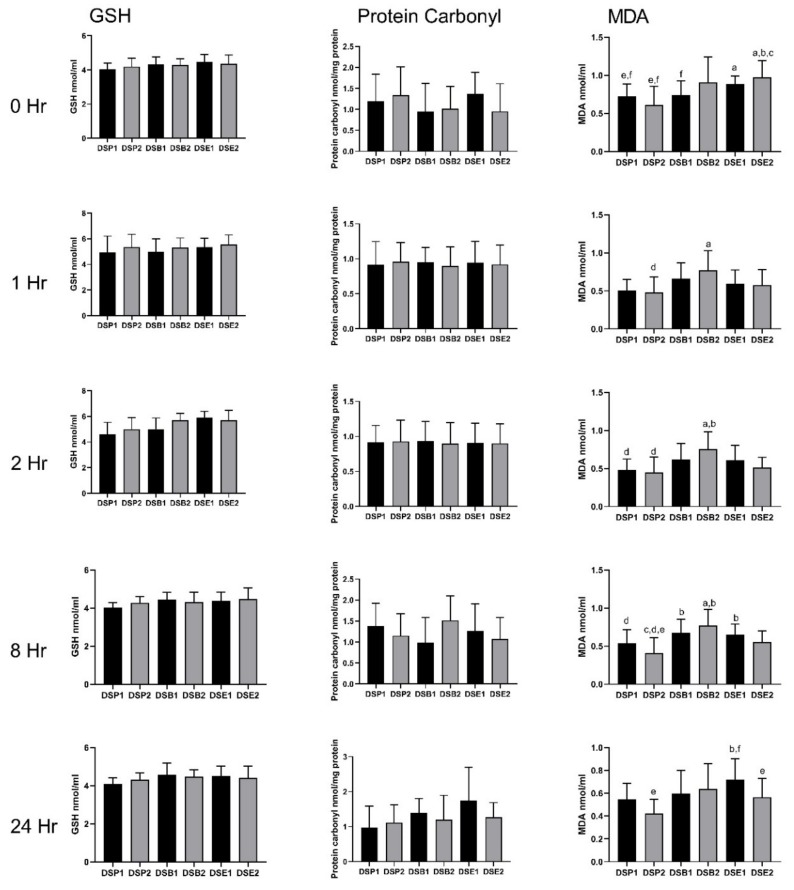
Comparisons of biomarkers of antioxidant defence and oxidative damage between the six treatments, at each time point, in the 14 participants. Means ± SD are presented. * ANOVA with repeated measures was performed. Gender and polyphenols consumption tertiles were added into the model as between subjects’ factor. Statistical significance was set at *p* ≤ 0.05. ^a^ Statistically significant difference with DSP1; ^b^ Statistically significant difference with DSP2; ^c^ Statistically significant difference with DSB1; ^d^ Statistically significant difference with DSB2; ^e^ Statistically significant difference with DSE1; ^f^ Statistically significant difference with DSE2.

Regarding the impact of the treatments on the antioxidant defence system, GSH level increased significantly compared to the baseline, 1 h after ingestion in all six treatments. This change in GSH ranged from 36.44% with DSE1 to 57.11% with DSP1 (Figure 5) and was maintained up to 8 h after ingestion. In parallel, 1 h after ingestion, the level of protein carbonyl was significantly reduced compared to baseline in all six treatments, from 87.50% with DSE2 to 93.57% with DSE1 (Figure 5). Eight hours after ingestion, the values were back to the baseline levels of the corresponding treatment. However, for both GSH and protein carbonyl, the values did not significantly differ between the treatments at any time point (Figure 6).

Malondialdehyde content was modified with DSP1, DSP2, DSE1 and DSE2 but not with DSB1 and DSB2 (both date seeds bread) (Figure 5). Briefly, with DSP1 and DSE1, a significant decrease compared to baseline was observed at 1 h after ingestion. This was in a more significant manner with DSE1, since the baseline value was significantly higher with DSE1 compared with DSP1. However, it was only transitory. Values with DSE1 and DSP1 were back to baseline levels, 2 h and 8 h after ingestion, respectively. By contrast, with higher doses of date seeds powder (DSP1) and extract (DSE2), the significant decrease reported at 1 h was extended up to 24 h, with again a more efficient impact of the extract compared to the powder (Figure 6).

## 4. Discussion

The prevalence of chronic diseases is alarming worldwide, and recent scientific and therapeutic progress has failed in curbing its growth. The use of natural and functional ingredients is a possible alternative that could contribute to protecting the populations against such diseases. Knowing the crucial role of oxidative stress in the development of chronic diseases, ingredients with antioxidant properties represent potent candidates for the development of functional food products. However, the bioavailability of the bioactive compounds from these ingredients will determine their real health potential in humans. Indeed, digestive, absorptive and metabolic processes are likely to affect the chemical structure of these compounds and consequently, their biological activity. Therefore, it is not enough to confirm the health properties in humans, a description of their bioavailability is also critical.

Date seeds, a local natural resource, are an excellent source of polyphenols [15]. Antioxidant properties have already been reported, both in vitro and in animals [16,17,28]. From our knowledge, this study is the first human trial to clearly show that date seeds powder, bread, and extract can be safely consumed by humans. A strengthening effect of the antioxidant defence system in the human body and protection against protein and lipids oxidative damages were observed up to 8 and 24 h after ingestion, respectively. The kinetics of the urine excretion of polyphenols metabolites, as well as the type of metabolites that were detected, support an implication of date seeds polyphenols. In the past, date seeds were traditionally incorporated in a coffee-like beverage and were consumed without any side effect ever being reported. Also, in two previous animal studies, the consumption of date seeds for several weeks did not alter the functions of organs, including heart, kidney, liver, and muscle [16,17,28]. Consequently, it is not surprising that no sign of toxicity has been detected in this present study. This supports a safe consumption by a human of a single dose of 0.5 g/kg bodyweight of date seeds powder, 360 g of 15% date seeds bread, and 60 mg/kg bodyweight of date seeds extract.

Besides, the corresponding amounts of date seeds products (date seeds powder solubilised in water, date seeds bread, and date seeds extract paste) were quite easily consumed by the subject. The total amount of polyphenols in each dose was much below the average estimated daily intake of polyphenols of the study sample, which is supporting the fact that doses were all realistic.

Similar to previous animal studies [16,17,28], the tested doses were able to strengthen the oxidative defence system and to protect against oxidative damages on protein and lipids. To date, only a few studies have explored and studied the effect of a single dose of polyphenols and results remain contradictory. An acute intake of a dose of grape/pomegranate pomace containing about 1.8 g of polyphenols was not able to clearly improve the oxidative status in adult subjects with abdominal obesity [29], while in another study, the consumption of 400 mL of a phenolic-rich beverage was related to reduced oxidative damages on protein and lipids [30].

**Figure 7:**
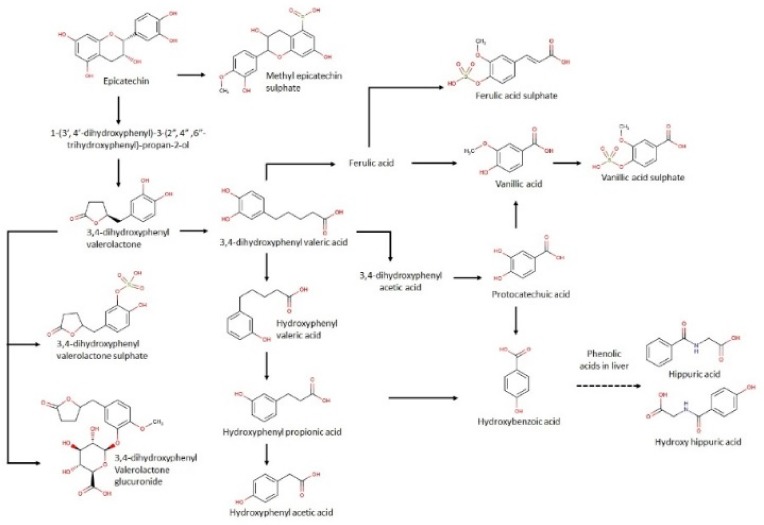
Proposed metabolic pathway of date seed polyphenols in human.

Overall, the kinetics of urine excretion of polyphenols is in agreement with previous results for other polyphenol sources such as cocoa [31] and red wine [32]. After consuming the six treatments of date seeds, 20 predominant metabolites that are previously reported with consumption of proanthocyanidins were observed in urine [33]. The results indicate a significant increase in levels of aromatic acids such as protocatechuic acid, hydroxybenzoic acid, vanillic acid, vanillic acid sulphate, and ferulic acid sulphate in the first three hours of consumption compared to the baseline. The levels of these aromatic compounds were sustained up to 24 h in the urine samples. Earlier studies from our group already reported the presence of protocatechuic acids and hydroxybenzoic acid in date seeds, which explains the immediate presence in urine [15]. It is noteworthy that these compounds are also released from microbial metabolism of catechins and protocatechuic acid, which explains their sustained release until 24 h in the study [33,34]. A brief illustration of the pathways of date seed gut metabolites is provided in Figure 7.

Vanillic acid is significantly increased at 0–3 h in comparison to baseline, which could be attributed to its formation in the liver by methylation [35]. A similar observation was reported by Gonthier et al. in their study with red wine powder supplementation in rats [32]. Liver metabolism can be similarly attributed to the increase in vanillic acid sulphate at 0–3 h after date seed consumption. A previous study with cocoa reports increases in ferulic acid but after 8 h of consumption [31], which indicates its formation through gut microbial metabolism from 3-hydroxyphenyl propionic acid. By contrast, here, the release of ferulic acid sulphate is happening in the urine immediately in the 0–3 h following consumption and is sustained until 24 h. An in vitro bioaccessibility study with digested date seed samples revealed transport of caffeoyl shikimic acids (ester of caffeic acid) across intestinal monolayer readily following 4 h incubation. Since caffeic acid is a precursor of ferulic acid, its formation in the liver in the initial hours cannot be ignored.

Other important proanthocyanidin metabolites that were identified are valerolactone compounds and other aromatic acids, such as 3-hydroxyphenyl acetic, propionic, and valeric acid along with 3–4 dihydroxyphenyl valeric acid and its sulphate, and hippuric and hydroxy hippuric acid. All of these metabolites are well documented with the consumption of other proanthocyanin sources such as grapeseed and cocoa [31,32]. The origin of these metabolites is in the colon by the action of gut microbes. Like the results of cocoa and grape seeds, a peak between the period of 3–8 h and a sustained release up to 24 h in urine were observed with date seed treatments. It is reported that hydroxyhippuric acid is formed from hydroxybenzoic acid in the liver and the metabolism of all the aromatic acids are interlinked [35], which is in agreement with the kinetics of these compounds in our study. Interestingly, the only conjugated catechin metabolite we found is methyl epicatechin sulphate. Gonthier et al. [34] suggested that the degree of polymerisation dictates the number of urinary metabolites observed. We have already reported on a high degree of polymerisation of date seed samples [21]. Phenyl valerolactones and phenyl valeric acids are well-reported metabolites of catechin and increased significantly across all date seed treatments and doses from 3–8 h following the ingestion of date seed [32,35].

The antioxidant effects that are reported in this study are most probably involving date seeds polyphenols and metabolites. First, such effects are typical of polyphenols. Then, an up-regulation of the body enzymatic defence system, like GSH, through the activation of transcription factors and the scavenging of free radicals through either a direct interaction with the radicals or the inhibition of enzymes involved in the free radicals’ production were described as some of the primary mechanisms by which polyphenols exert their antioxidant properties [2]. Finally, the kinetics of the urine excretion of polyphenols metabolites and their type are further supporting the implication of polyphenols from date seeds. Indeed, while GSH was increasing and oxidative damages were reduced, an abundance of polyphenols was found in the urine, up to 8 h after consumption. Also, the trend to a decrease of GSH between 8 and 24 h occurred in parallel with the less intense urine excretion of polyphenols.

Interestingly, up to 8 h, the urine polyphenols are similar to those identified after in vitro intestinal absorption [21] and are among the compounds with the highest antioxidant properties [34,36,37]. Between 8 and 24 h, mainly metabolites with lower antioxidant power and resulting from the gut microbiota metabolism [34,38,39] were released in the urine. This is in agreement with the less extensive antioxidant effect of date seeds products that was observed between 8 and 24 h. Further, there is a clear, tight relationship between the antioxidant power, its underlying mechanism, and the chemical structure of the polyphenolic compounds [40]. Notably, a more significant number of reactive sites-binding free radicals is enhancing the ability to scavenge free radicals. Interestingly, this is the case of the polyphenols detected after 8 h compared to those released before. This can explain the prolonged protective effect against protein and lipids oxidative damages between 8 and 24 h.

Noticeably, the consumption of date seeds bread did decrease the level of protein carbonyl but not the level of MDA, suggesting that polyphenols from date seeds bread would bind more proteins than lipids to protect against oxidative damages. It is well known that the release of glucose in the blood is inducing an increased production of free radicals. These later were shown to increase the level of advanced glycation end products, which in turn would raise the level of protein carbonyls [41]. The carbohydrates content of date seeds bread is higher compared to the date seeds powder and extract. Consequently, the consumption of the bread itself would generate an additional amount of protein carbonyl groups, reinforcing the level of oxidative stress and attracting polyphenols. A recent work which was aiming at identifying the interactions between polyphenols and proteins highlighted that sixty-five percent of the polyphenols had at least 10 reported polyphenol–protein interactions, with flavones, hydroxybenzoic acids, and alkylphenols being the sub-classes with the largest number of interactions with proteins [42]. Being linked to proteins, polyphenols from date seeds bread could be less available to protect lipids from oxidation. Besides, a protective role from polysaccharides found in bread, against protein glycation, could not be excluded since such an effect was reported with other natural products rich in polyphenols [43,44].

Polyphenols are preventing the development of chronic diseases in different other ways. This includes a lipid-lowering effect and a modulation of the blood glucose level [45,46,47]. However, the antioxidant activity of the polyphenols was commonly identified as a mediator. In this present work, even though date seeds polyphenols were able to positively influence the oxidative status of the subjects, no significant change of the lipid profile and the glycemia was observed. The decrease in blood biochemical parameters is observed with long term consumption. Indeed, a high number of clinical trials reported a significant impact of polyphenols consumption on lipid and glucose levels after several weeks [48]. However, a recent meta-analysis supports a lack of effect on the lipid profile of famous polyphenols compounds like resveratrol or quercetin [49,50]. With all of this taken together, our results could have been expected.

It should be noticed that the analysis here included only 14 subjects. However, this sample size is within the range of several subjects being included in similar clinical trials. In this study, a qualitative approach was used to study the urine excretion of the polyphenol metabolites. Urine analysis was not performed against metabolites standards. Therefore, the results are reflecting the total excretion of polyphenols metabolites and the presence of specific compounds but without further quantification. This is preventing us from highlighting the probable differences between the date seeds products. However, this allows us to conclude about the implication of date seeds polyphenols in the expression of the antioxidant effects after a single dose of date seeds powder, bread, or extract and, in this way, to satisfy the purpose of the study. Finally, doses were tested only one time in each subject. Although the experimental conditions were carefully controlled for all subjects and for each treatment, it is hard to conclude about a good level of reliability of our results.

The interaction of undigested polyphenols with microbiota was not considered here. This should be studied to provide a complete overview of the bioavailability of date seeds polyphenols. Indeed, an interaction between gut microorganisms and polyphenols is recognized, with polyphenols influencing the growth of the microorganisms and the gut microbiota print and gut microorganisms metabolizing the “undigestible” polyphenolic compounds with the release of metabolites likely to be absorbed and to exert biological effects in the human body.

This work highlighted the antioxidant effect of a single dose of date seeds products (powder, bread and extract) in humans. This is in favour of a high potential of date seeds products as functional ingredients, knowing that further studies testing a longer-term consumption of date seeds products should be conducted to confirm our results.

## Figures and Tables

**Table 1:** Description of the date seed treatments.

Treatment Number	Product	Dose	Polyphenols Content of the Product (g/kg)	Total Average Dose of Polyphenols * (g)
DSP1	Date seed powder	0.25 g/kg body weight	49.75	0.74
DSP2	Date seed powder	0.5 g/kg body weight	99.5	1.48
DSB1	Date seed pita bread	10% date seed powder, 6 loaves of 60 g each	13.48	1.79
DSB2	Date seed pita bread	15% date seed powder, 6 loaves of 60 g each	26.96	2.69
DSE1	Date seed extract	30 mg/kg body weight	41.11	0.075
DSE2	Date seed extract	60 mg/kg body weight	82.22	0.15

* The average body weight of the sample was considered for calculation of the average dose. DSP: Date Seed Powder; DSB: Date Seed Bread; DSE: Date Seed Extract.

**Table 2:** Characteristics of the participants (n = 14).

	Total (n = 14)	Female (n = 8)	Male (n = 6)
Age (y)	25.43 ± 5.11	26.75 ± 4.50	23.67 ± 5.75
Height (cm)	163.21 ± 10.44	156.14 ± 5.63	172.65 ± 7.20 *
Weight (kg)	59.43 ± 6.97	54.81 ± 4.02	65.58 ± 4.92 *
BMI (kg/m^2^)	22.32 ± 1.79	22.51 ± 1.58	22.07 ± 2.16
Polyphenols consumption (g/d)	3.09 ± 1.93	3.58 ± 2.35	2.27 ± 0.89

Means ± standard deviation (SD) are presented. * ANOVA was used to assess gender differences. Statistical significance set at *p* ≤ 0.05. BMI: body mass index.

**Table 3:** Biochemical parameters, for each treatment, at baseline and 24 h after treatment ingestion in the 14 participants.

	Glucose (mg/dl)	Total Cholesterol (mg/dl)	LDL-Cholesterol (mg/dl)	HDL-Cholesterol (mg/dl)	Triglycerides (mg/dl)
**DSP1**					
Baseline	91.07 ± 6.68	171.70 ± 25.46	107.66 ± 24.19	45.16 ± 10.16	72.49 ± 21.70
24 h	93.99 ± 7.01	169.77 ± 25.04	104.55 ± 24.92	46.56 ± 9.61	78.27 ± 30.76
**DSP2**					
Baseline	86.32 ± 12.98	169.58 ± 21.79	98.83 ± 25.93	46.71 ± 16.74	71.50 ± 25.72
24 h	91.01 ± 7.31	168.94 ± 21.09	98.91 ± 21.00	48.12 ± 13.70	76.04 ± 17.62
**DSB1**					
Baseline	87.67 ± 8.81	160.74 ± 27.68	97.07 ± 22.96	48.15 ± 17.35	71.95 ± 23.81
24 h	88.84 ± 9.64	162.61 ± 29.94	92.99 ± 23.84	45.98 ± 15.76	71.20 ± 15.08
**DSP2**					
Baseline	85.13 ± 9.85	165.47 ± 24.49	99.18 ± 23.44	47.07 ± 15.36	75.91 ± 22.74
24 h	88.26 ± 11.38	169.26 ± 31.13	97.12 ± 25.40	46.39 ± 13.42	78.45 ± 19.06
**DSE1**					
Baseline	84.78 ± 8.46	158.45 ± 27.20	92.54 ± 24.56	45.60 ± 15.92	80.94 ± 37.63
24 h	89.56 ± 5.41 *	168.28 ± 29.16 *	100.16 ± 25.99 *	47.47 ± 14.41	79.99 ± 28.65
**DSE2**					
Baseline	84.74 ± 6.45	164.69 ± 20.28	88.17 ± 24.96	46.61 ± 19.31	65.19 ± 19.62
24 h	83.70 ± 13.05	166.85 ± 23.81	91.25 ± 24.54	43.97 ± 16.25	84.37 ± 39.80

Means ± SD are presented. * Paired t-test was used to compare values at baseline and 24 h. Statistical significance set at *p* ≤ 0.05. DSP: Date Seed Powder; DSB: Date Seed Bread; DSE: Date Seed Extract.

**Table 4:** Biomarkers of tissue function and injury, for each treatment, at baseline and 24 h after treatment ingestion in the 14 participants.

	Creatinine (µmol/l)	Albumin (g/dl)	Urea (mmol/l)	Creatine Kinase (U/dl)	Lactate Dehydrogenase (U/l)	GGT (U/l)	AST (U/l)	ALT (U/l)	ALP (U/l)
**DSP1**									
Baseline	61.70 ± 13.54	4.67 ± 0.24	4.22 ± 1.13	94.70 ± 47.47	165.27 ± 56.72	14.26 ± 6.86	18.05 ± 4.05	14.72 ± 7.12	57.01 ± 14.01
24 h	62.30 ± 12.70	4.64 ± 0.29	3.91 ± 0.80	82.08 ± 35.26 *	136.77 ± 14.11	14.03 ± 6.84	16.57 ± 3.59	14.93 ± 8.81	59.23 ± 12.35
**DSP2**									
Baseline	56.90 ± 17.55	4.31 ± 0.67	4.06 ± 1.27	83.69 ± 44.95	126.00 ± 32.23	12.93 ± 7.76	15.85 ± 2.46	13.83 ± 6.08	55.71 ± 16.25
24 h	60.93 ± 13.61	4.64 ± 0.36	4.1186 ± 1.10	77.19 ± 27.98	115.78 ± 24.65	15.04 ± 8.54 *	16.25 ± 3.26	14.26 ± 6.05	62.68 ± 16.31 *
**DSB1**									
Baseline	57.99 ± 16.71	4.50 ± 0.76	3.91 ± 1.06	83.87 ± 42.99	120.10 ± 17.04	13.93 ± 11.64	15.82 ± 3.50	13.06 ± 5.13	57.71 ± 19.43
24 h	59.31 ± 15.53	4.31 ± 0.66	3.74 ± 0.95	74.85 ± 30.25	95.77 ± 33.12 *	14.00 ± 10.68	14.64 ± 2.70	12.01 ± 4.90	57.32 ± 15.74
**DSB2**									
Baseline	58.55 ± 16.69	4.40 ± 0.60	3.82 ± 1.03	87.82 ± 47.43	124.77 ± 27.93	13.35 ± 8.11	15.64 ± 4.30	12.61 ± 6.85	57.42 ± 16.82
24 h	60.25 ± 16.88	4.38 ± 0.72	3.75 ± 0.83	79.81 ± 33.55	98.17 ± 23.88 *	14.13 ± 9.26	15.13 ± 4.83	12.72 ± 8.17	57.78 ± 16.12
**DSE1**									
Baseline	60.84 ± 15.33	4.31 ± 0.62	3.83 ± 1.24	82.75 ± 29.33	125.04 ± 79.58	12.74 ± 8.37	15.94 ± 6.16	11.89 ± 7.02	54.79 ± 16.10
24 h	60.56 ± 14.71	4.66 ± 0.39 *	3.64 ± 0.86	79.64 ± 27.69	106.56 ± 25.37	14.01 ± 7.76	15.18 ± 2.94	11.89 ± 6.26	56.89 ± 12.55
**DSE2**									
Baseline	51.57 ± 16.15	4.20 ± 0.61	3.47 ± 1.31	77.84 ± 39.22	110.04 ± 35.85	11.33 ± 6.26	13.71 ± 3.53	11.23 ± 5.30	48.96 ± 17.24
24 h	56.91 ± 17.40	4.42 ± 0.57	3.76 ± 1.02	69.80 ± 27.41	107.91 ± 35.63	13.63 ± 10.47	13.09 ± 3.60	12.01 ± 6.75	56.99 ± 15.75

Means ± SD are presented. * Paired t-test was used to compare values at baseline and 24 h. Statistical significance set at *p* ≤ 0.05. DSP: Date Seed Powder; DSB: Date Seed Bread; DSE: Date Seed Extract; GGT: gamma-glutamyltransferase

**Table 5:** Metabolites of date seed polyphenols identified in the urine by ultra-performance liquid chromatography-quadrupole-time-of-flight Mass spectrometry (UPLC-QTOF-MS/MS) analysis following ingestion of date seed treatments.

Compound	Formula	Pseudo-molecular ion (M-H) Exact	Retention Time (min)	Score	Error
Hydroxybenzoic acid 1	C_7_ H_6_ O_3_	137.0244	3.090	99.49 ± 0.05	1.43 ± 2.45
Hydroxybenzoic acid 2	C_7_ H_6_ O_3_	137.0244	4.464	97.58 ± 0.84	2.86 ± 0.10
Protocatechuic acid 1	C_7_ H_6_ O_4_	153.0193	4.640	96.98 ± 1.06	3.73 ± 3.83
Protocatechuic acid 2	C_7_ H_6_ O_5_	153.0193	5.352	95.31 ± 2.06	1.64 ± 2.64
Vanillic acid 1	C_8_ H_8_ O_4_	167.035	3.300	95.39 ± 3.67	1.66 ± 3.93
Vanillic acid 2	C_8_ H_8_ O_4_	167.035	4.735	93.64 ± 3.29	0.23 ± 3.31
Vanillic acid sulphate	C_8_ H_8_ O_7_ S	246.9918	3.368	93.26 ± 2.10	2.19 ± 1.98
Ferulic acid sulphate	C_10_ H_6_ O_7_ S	273.0074	4.618	93.78 ± 2.44	2.31 ± 2.18
Methyl epicatechin sulphate	C_16_ H_6_ O_9_ S	383.0442	5.623	89.13 ± 3.55	0.01 ± 2.04
3,4-dihydroxyphenyl valerolactone	C_11_ H_12_ O_4_	207.0663	5.250	97.03 ± 1.76	2.56 ± 0.57
3,4-dihydroxyphenyl valerolactone glucuronide	C_17_ H_20_ O_10_	383.0984	5.126	97.30 ± 2.47	3.95 ± 2.44
3,4-dihydroxyphenyl valerolactone sulphate	C_11_ H_12_ O_7_ S	287.0231	5.273	96.60 ± 2.40	1.50 ± 0.89
3-hydroxyphenyl acetic acid	C_8_ H_8_ O_3_	151.0401	6.006	91.47 ± 0.83	2.95 ± 2.55
3-hydroxyphenyl propionic acid	C_9_ H_10_ O_3_	165.0557	4.968	92.76 ± 0.78	1.89 ± 2.80
3-hydroxyphenyl valeric acid	C_11_ H_14_ O_3_	193.087	6.979	90.11 ± 0.63	2.65 ± 3.78
3,4-dihydroxyphenyl valeric acid	C_11_ H_14_ O_4_	209.0819	10.407	95.02 ± 1.11	0.56 ± 3.56
3,4-dihydroxyphenyl valeric acid sulphate	C_11_ H_14_ O_7_ S	289.0387	10.863	92.48 ± 4.85	0.91 ± 0.46
Hippuric acid	C_9_ H_9_ NO_3_	178.051	4.921	98.47 ± 0.28	1.87 ± 1.86
Hydroxy hippuric acid 1	C_9_ H_9_ NO_4_	194.0459	3.703	95.91 ± 1.71	0.58 ± 0.76
Hydroxy hippuric acid 2	C_9_ H_9_ NO_4_	194.0459	4.505	94.49 ± 0.78	3.28 ± 3.77
